# Supplementary material for: Polyketide synthase gene clusters in the ericoid mycorrhizal fungus Oidiodendron maius: genome-wide prediction, expression profiling, and functional characterization of knockout mutants
Source: Front Plant Sci. 2026 Sep 16;17:1924430. doi: 10.3389/fpls.2026.1924430 (PMC13623903; doi:10.3389/fpls.2026.1924430)
Supplement: Supplementary file 1 [file SupplementaryFile1.zip › Supplementary Materials/Supplementary Folder 1.zip!/Oidma1_AssemblyScaffolds_Repeatmasked/knownclusterblast/cluster13/jgi.p_Oidma1_39623_mibig_hits.html]

| MIBiG Protein | Description | MIBiG Cluster | MiBiG Product | % ID | % Coverage | BLAST Score | E-value |
| --- | --- | --- | --- | --- | --- | --- | --- |
| ADY16699.1 | TqaJ | BGC0001142 | Tryptoquialanine\_biosynthetic\_gene\_cluster | 29 | 87.2798434442 | 173 | 6.4e-43 |
| CAH10123.1 | putative\_transporter | BGC0000268 | Sch47554\_/\_Sch47555\_biosynthetic\_gene\_cluster | 28 | 96.6731898239 | 142 | 1.2e-33 |
| CAF31575.1 | putative\_kanamycin\_efflux\_protein | BGC0000705 | Kanamycin\_biosynthetic\_gene\_cluster | 30 | 85.1272015656 | 139 | 1.3e-32 |
| CAF60521.1 | putative\_efflux\_protein | BGC0000704 | Kanamycin\_biosynthetic\_gene\_cluster | 30 | 85.1272015656 | 139 | 1.3e-32 |
| BAV16993.1 | putative\_transporter | BGC0001384 | Saprolmycin\_biosynthetic\_gene\_cluster | 29 | 82.5831702544 | 138 | 1.8e-32 |
| WP\_052390548.1 | hypothetical\_protein | BGC0001308 | Simocyclinone\_D9\_/\_simocyclinone\_D10\_/\_simocyclinone\_D11 | 29 | 86.1056751468 | 134 | 2.5e-31 |
| AGO50605.1 | transporter | BGC0000229 | Grincamycin\_biosynthetic\_gene\_cluster | 27 | 91.3894324853 | 134 | 3.3e-31 |
| AAD13557.1 | LanJ | BGC0000239 | Landomycin\_biosynthetic\_gene\_cluster | 28 | 84.5401174168 | 131 | 3.7e-30 |
| AFU65888.1 | DacR2 | BGC0000216 | Dactylocycline\_biosynthetic\_gene\_cluster | 27 | 91.3894324853 | 130 | 6.3e-30 |
| AAF00219.1 | transporter | BGC0000276 | Urdamycin\_biosynthetic\_gene\_cluster | 25 | 95.3033268102 | 126 | 6.9e-29 |
| ADG86335.1 | transporter | BGC0000190 | A-74528\_biosynthetic\_gene\_cluster | 26 | 89.8238747554 | 123 | 7.6e-28 |
| AHW57792.1 | PgaJ3 | BGC0000262 | Rabelomycin\_biosynthetic\_gene\_cluster | 25 | 85.518590998 | 121 | 2.2e-27 |
| ABC87523.1 | putative\_drug\_efflux\_transporter | BGC0001011 | Meridamycin\_biosynthetic\_gene\_cluster | 28 | 86.1056751468 | 106 | 7.4e-23 |
| CAQ64674.1 | putative\_MFS-type\_efflux\_protein | BGC0000087 | Lasalocid\_biosynthetic\_gene\_cluster | 27 | 87.2798434442 | 103 | 8.2e-22 |
| ADE34501.1 | SsfR | BGC0000269 | SF2575\_biosynthetic\_gene\_cluster | 24 | 93.1506849315 | 86 | 1.4e-16 |
| AEA60653.1 | transporter | BGC0000892 | Caryoynencin\_biosynthetic\_gene\_cluster | 23 | 87.6712328767 | 80 | 7.4e-15 |
| AAP69589.1 | putative\_transmembrane\_efflux\_protein | BGC0000226 | Gilvocarcin\_biosynthetic\_gene\_cluster | 24 | 103.522504892 | 75 | 2.4e-13 |
| CAD18978.1 | putative\_transport\_protein | BGC0000847 | Thienamycin\_biosynthetic\_gene\_cluster | 24 | 78.8649706458 | 67 | 5e-11 |
| EAU36741.1 | conserved\_hypothetical\_protein | BGC0000292 | Acetylaranotin\_biosynthetic\_gene\_cluster | 23 | 52.6418786693 | 50 | 8.2e-06 |
